# *Helicobacter pylori* Dampens HLA-II Expression on Macrophages via the Up-Regulation of miRNAs Targeting CIITA

**DOI:** 10.3389/fimmu.2019.02923

**Published:** 2020-01-08

**Authors:** Gaia Codolo, Marta Toffoletto, Francesco Chemello, Sara Coletta, Gemma Soler Teixidor, Greta Battaggia, Giada Munari, Matteo Fassan, Stefano Cagnin, Marina de Bernard

**Affiliations:** ^1^Department of Biology, University of Padua, Padua, Italy; ^2^CRIBI Biotechnology Center, University of Padua, Padua, Italy; ^3^Istituto Oncologico Veneto (IRCCS), Padua, Italy; ^4^Department of Medicine, University of Padua, Padua, Italy; ^5^CIR-Myo Myology Center, University of Padua, Padua, Italy

**Keywords:** *Helicobacter pylori*, miRNA, macrophages, antigen presentation, gastric cancer

## Abstract

Macrophages have a major role in infectious and inflammatory diseases, and the available data suggest that *Helicobacter pylori* persistence can be explained in part by the failure of the bacterium to be killed by professional phagocytes. Macrophages are cells ready to kill the engulfed pathogen, through oxygen-dependent and -independent mechanisms; however, their killing potential can be further augmented by the intervention of T helper (Th) cells upon the specific recognition of human leukocyte antigen (HLA)-II–peptide complexes on the surface of the phagocytic cells. As it pertains to *H. pylori*, the bacterium is engulfed by macrophages, but it interferes with the phagosome maturation process leading to phagosomes with an altered degradative capacity, and to megasomes, wherein *H. pylori* resists killing. We recently showed that macrophages infected with *H. pylori* strongly reduce the expression of HLA-II molecules on the plasma membrane and this compromises the bacterial antigen presentation to Th lymphocytes. In this work, we demonstrate that *H. pylori* hampers HLA-II expression in macrophages, activated or non-activated by IFN-γ, by down-regulating the expression of the class II major histocompatibility complex transactivator (CIITA), the “master control factor” for the expression of HLA class II genes. We provided evidence that this effect relies on the up-regulation of let-7f-5p, let-7i-5p, miR-146b-5p, and -185-5p targeting CIITA. MiRNA expression analysis performed on biopsies from *H. pylori*-infected patients confirmed the up-regulation of let-7i-5p, miR-146b-5p, and -185-5p in gastritis, in pre-invasive lesions, and in gastric cancer. Taken together, our results suggest that specific miRNAs may be directly involved in the *H. pylori* infection persistence and may contribute to confer the risk of developing gastric neoplasia in infected patients.

## Introduction

The prevalence of infection by *Helicobacter pylori* is declining in the developed world, but the bacterium remains a major cause of morbidity and mortality worldwide. It invariably causes active chronic gastritis that, in many people, may be clinically silent throughout life, but in a significant minority, it results in gastroduodenal diseases, especially peptic ulcer disease, atrophic gastritis, gastric mucosa-associated lymphoid tissue (MALT) lymphoma, and gastric cancer (GC) (1). Although it is no longer the most common cancer worldwide, GC remains the second leading cause of cancer-related mortality worldwide and the most prevalent cancer in Eastern Asia (2). GCs are classified into two major histological types: diffuse and intestinal; these two types of cancer seem to follow different precancerous processes and show clinical and epidemiologic differences, but *H. pylori* infection is the strongest risk factor for the development of both tumor types (3). It is believed that *H. pylori* contributes to GC development by direct action of its virulence factors and indirectly by initiation and maintenance of a longstanding inflammatory insult of the gastric mucosa (4). Following bacterial infection, inflammation is elicited with an important infiltration of the gastric mucosa by polymorphonuclear cells (PMNs) and mononuclear phagocytes; subsequently, T helper (Th) cells, mainly belonging to the Th1 and Th17 subsets, are also recruited to the gastric mucosa (5, 6). To deal with this robust immune response, *H. pylori* has developed several strategies to escape recognition and to dampen immune responses (7); as a result, the bacterium can persist lifelong in the host, thus leading to the chronicization of the inflammatory status that paves the way for the development of more severe gastric disorders.

Macrophages have a major influence on inflammation, and the available data suggest that *H. pylori* persistence can be explained in part by the failure of the bacterium to be killed by professional phagocytes (8, 9).

Macrophages are cells ready to kill the engulfed pathogens, through oxygen-dependent and -independent mechanisms. However, their killing potential can be further augmented by the intervention of Th cells. The latter, upon the specific recognition of HLA-II–peptide complexes on the surface of the phagocytic cells, release IFN-γ. The cytokine promotes the production of inflammatory mediators, the antigen presentation by phagocytes, and the microbial killing.

As it pertains to *H. pylori*, the bacterium is engulfed by macrophages, but it interferes with the phagosome maturation process leading to phagosomes with an altered degradative capacity, and to megasomes, wherein *H. pylori* resists killing (9–11). Moreover, macrophages infected with *H. pylori* strongly reduce the exposure of HLA-II molecules on the plasma membrane compromising the bacterial antigen presentation toward Th lymphocytes (12). Ultimately, this would impair the local proliferation of Th cells and the possibility for them to exert the effector function, including the activation of the killing potential of macrophages.

Although this evidence supports the notion that macrophages may represent a survival niche for the bacterium in the stomach and emphasize the key role of these professional phagocytes in the *H. pylori*-related disorders, mechanism(s) by which the bacterium manipulates the antigen-presentation pathway of macrophages remained an issue to be addressed. We and others have demonstrated that *H. pylori* modifies the expression of microRNAs (miRNAs) involved in the regulation of inflammatory response and immune host response to the bacterium (12–15). These observations opened the possibility that the modulation of specific miRNAs could be responsible for the observed reduction of HLA-II expression in infected macrophages.

In this work, we demonstrate that *H. pylori* induces the expression of let-7f-5p, let-7i-5p, miR-146b-5p, and -185-5p, in macrophages. By targeting the class II major histocompatibility complex transactivator (CIITA), the “master control factor” for the expression of HLA class II genes, these miRNAs down-regulate the expression of CIITA and, in turn, that of HLA class II genes and proteins. Among those, let-7i-5p, miR-146b-5p, and -185-5p are essential for regulating HLA-II in *H. pylori*-infected macrophages. A meta-analysis approach and the quantification of miRNA expression in the gastric mucosa of *H. pylori*-infected patients supported the notion that let-7i-5p, miR-146b-5p, and -185-5p might be involved in the onset of gastritis and the subsequent stages of pre-neoplastic and neoplastic conditions.

## Results

### *H. pylori* Interferes With HLA-II Expression in Macrophages

**Figure 1:**
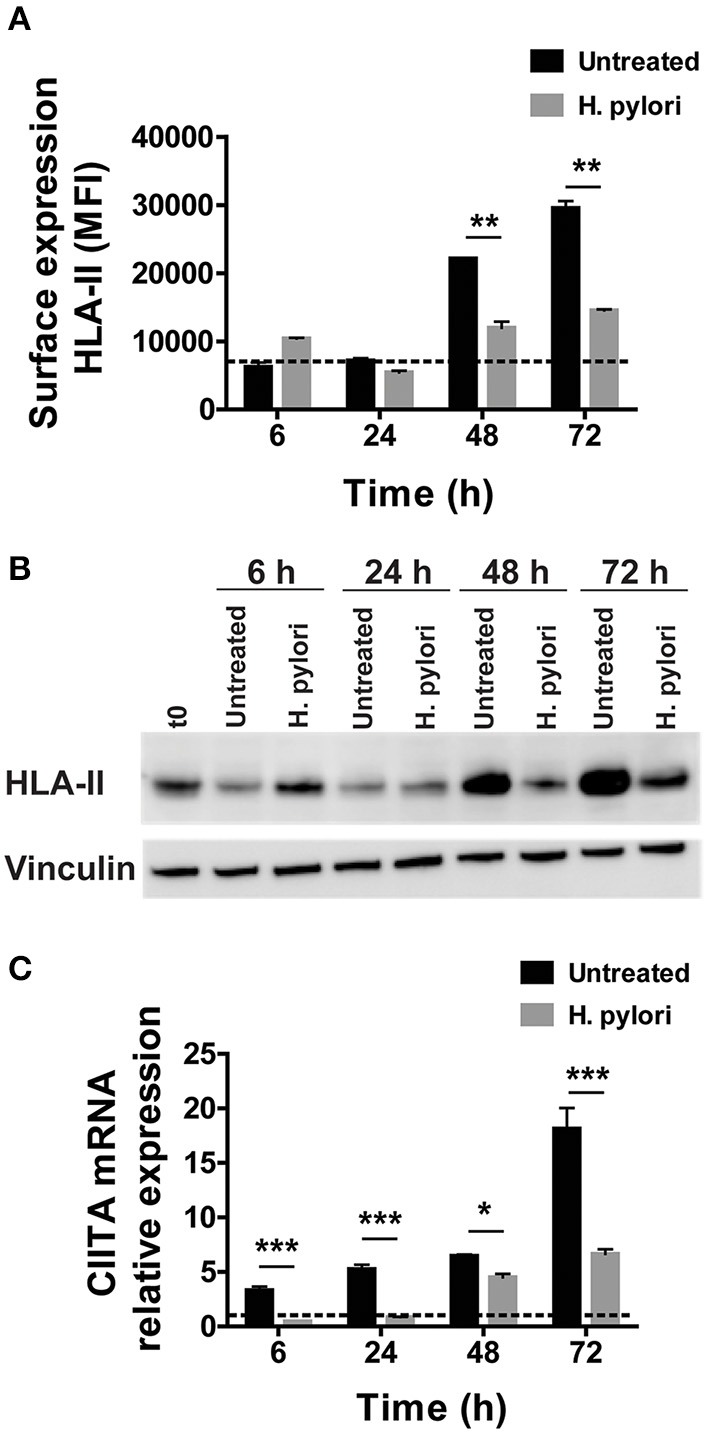
Expression and transcriptional modulation of HLA-II in macrophages infected by *H. pylori*. **(A)** Surface expression of HLA-II protein in human macrophages infected by *H. pylori* (MOI 10) for 6, 24, 48, and 72 h. Data are expressed as median fluorescence intensity (MFI) ± SEM of five independent experiments. **(B)** HLA-II total cell content evaluated by Western blot in macrophages infected as in **(A)**. Blot refers to a representative of three independent experiments. **(C)** Relative expression of mRNA encoding CIITA in macrophages infected as in **(A)**. Data were normalized to an endogenous reference gene (β-actin). Values at T_0_ cells were taken as reference and set as 1 and the expression levels for treated cells were relative to the expression of T_0_ cells. Data are expressed as mean ± SEM of five independent experiments. Significance was determined by Student's *t*-test. *p < 0.05; **p < 0.01; and ***p < 0.001.

We have previously shown that a 48-h infection of human macrophages by *H. pylori* leads to a reduction of HLA-II molecules on macrophage surface (12). To confirm this evidence, we performed a time-course analysis, extending the time of infection to 72 h. As shown in Figure 1A, control cells maintained the amount of HLA-II on the plasma membrane at a basal level until 48 h of culture; at this time point, the number of exposed molecules doubled and further increased at 72 h. We interpreted this trend as a consequence of the replacement of the medium containing M-CSF with a medium deprived of the growth factor at the beginning of the experiment. In accordance, after removing M-CSF, macrophages progressively acquired an M1-like profile with high expression of CD86 and low expression of CD206 (Supplementary Figure 1). In infected cells, the amount of HLA-II expressed on the cell surface was comparable to that of uninfected ones during the first 24 h of infection; at 48 h, it increased but reached only 60% of controls. Seventy-two hours after the addition of bacteria to the cells, HLA-II was 50% lower than in uninfected cells, despite the fact that extracellular bacteria disappeared within a few hours after the infection. Moreover, while the infection also maintained low the expression of CD86, it increased that of CD206 (Supplementary Figure 1), in accordance with previous data (16).

We reasoned that this effect could rely either on a hindrance to the export of the presenting complex or on a blockage of its synthesis. We exposed macrophages to the bacterium, and we evaluated the HLA-II content in cell lysates. Similar to what we observed in terms of protein expressed on the plasma membrane of macrophages, after an initial small increase at 6 h, the total content of HLA-II in infected cells regained the basal level at 24 h, before rising again at 48 h and even more at 72 h. Nevertheless, at this time frame, the difference between infected and uninfected cells was impressive (Figure 1B). This observation ruled out any interference in the trafficking of HLA-II from the ER/Golgi toward the plasma membrane and opened the possibility that the bacterial infection affected HLA-II gene expression. The latter is finely tuned by the intervention of several transcription factors (17), but the master regulator is the CIITA (18). CIITA is expressed constitutively in antigen-presenting cells, including dendritic cells, macrophages, and B cells and is inducible in most other cell types (19). The absence of CIITA prevents the expression of HLA-II genes (20–22). In agreement with the time-dependent increase of HLA-II, the mRNA expression for CIITA progressively augmented in control cells; on the contrary, bacterial infection kept the expression of mRNA for CIITA to a basal level until 48 h, when it started to slightly increase, reaching a value at 72 h, which was less than half of that in control cells (Figure 1C).

### *H. pylori* Interferes With IFN-γ-Induced HLA-II Expression in Macrophages

The effect of *H. pylori* infection on HLA-II cell content and exposure as well as on CIITA mRNA expression was evidenced in cells that, for a change in culture condition (removal of M-CSF), progressively increased the expression of both the regulator and the antigen-presenting molecule. To exclude that what we were observing was affected by the experimental condition, we exposed M-CSF-differentiated macrophages with IFN-γ for 6 h before infecting or not the cells with the bacterium for 48 h. IFN-γ is a potent inducer of HLA-II expression and CIITA is the essential coactivator for both constitutive and IFN-γ-inducible HLA-II expression (18, 23, 24). The time frame of 6 h was the minimum time to appreciate the cytokine-induction of HLA-II expression on the surface of macrophages (data not shown).

**Figure 2:**
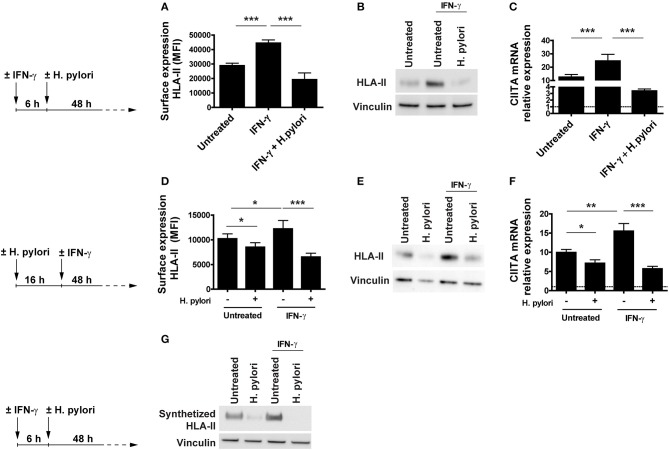
Expression and transcriptional modulation of HLA-II in IFN-γ-activated macrophages infected by *H. pylori*. **(A)** Surface expression of HLA-II protein in human macrophages activated for 6 h with 20 ng/ml IFN-γ before the infection with *H. pylori* (MOI 10) for 48 h. Data are expressed as MFI ± SEM of three independent experiments, performed with cells from three different donors. **(B)** HLA-II total cell content in macrophages infected as in **(A)**, evaluated by Western blot. Blot refers to a representative of three independent experiments, performed with cells from three different donors. **(C)** Relative expression of mRNA encoding CIITA in macrophages activated as in **(A)**. Data were normalized to an endogenous reference gene (β-actin). Values at T_0_ cells were taken as reference and set as 1 (dotted line); the expression levels for treated cells were relative to the expression of T_0_ cells. Data are expressed as mean ± SEM of three independent experiments, performed with cells from three different donors. **(D)** Surface expression of HLA-II protein in human macrophages infected for 16 h with *H. pylori* (MOI 10) and treated with 20 ng/ml IFN-γ for 48 h. Data are expressed as MFI ± SEM of three independent experiments, performed with cells from three different donors. **(E)** HLA-II total cell content in macrophages treated as in **(D)**, evaluated by Western blot. Blot refers to a representative of three independent experiments, performed with cells from three different donors. **(F)** Relative expression of mRNA encoding CIITA in macrophages activated as in **(D)**. Data were normalized to an endogenous reference gene (β-actin). Values at T_0_ cells were taken as reference and set as 1 (dotted line); the expression levels for treated cells were relative to the expression of T_0_ cells. Data are expressed as mean ± SEM of three independent experiments, performed with cells from three different donors. **(G)** HLA-II synthetic rate in macrophages treated as in **(A)**. At the end of stimulation, macrophages were labeled with the methionine analog AHA (L-Azidohomoalanine) for 2 h. Cells were lysed, and newly synthetized proteins were tagged with biotin alkyne. Proteins were pulled down with neutravidin agarose resin, processed for Western blot and developed for HLA-II. Blot refers to a representative of three independent experiments, performed with cells from three different donors. Significance was determined by Student's *t*-test. *p < 0.05; **p < 0.01; and ***p < 0.001.

Results illustrated in Figure 2A reveal that, while the activation of macrophages with the cytokine increased the expression of HLA-II on the plasma membrane with respect to unstimulated cells, the infection with *H. pylori* completely abrogated the effect. Moreover, the effect of *H. pylori* on HLA-II was so strong that the expression of HLA-II in IFN-γ-treated cells was maintained at a level lower than that in untreated cells (*p* ≤ 0.05) (Figure 2A). The same pattern was reproduced with the total cell content of HLA-II and with the expression of mRNA for CIITA (Figures 2B,C). It is worthy to note that the expression profile of CIITA mirrored the lower expression of HLA-II in IFN-γ-treated cells with respect to untreated cells (*p* ≤ 0.01) (Figure 2C).

IFN-γ is the signature cytokine of the Th1-polarized immune response, which features the *H. pylori*-infected gastric mucosa of humans (6, 25) and is the major activator of macrophages.

Based on our data, which supported the notion that the bacterium might inhibit the activity of IFN-γ *in vivo*, we were curious about whether *H. pylori* was able to hamper the effect of IFN-γ even in macrophages already infected with *H. pylori* since they are expected to present bacterial antigens to Th1 cells and become activated by the cytokine. Macrophages were infected 16 h with the bacterium and then exposed to IFN-γ. After 48 h, HLA-II exposed on the plasma membrane and its total cell content, as well as the expression of the mRNA for CIITA were determined. As above, *H. pylori* infection interfered with the IFN-γ signaling, which is essential for augmenting the antigen presentation capacity of macrophages (Figures 2D–F). Even in this case, the modulation of the expression of both HLA-II protein and CIITA mRNA was concordant in infected cells and the effect was more pronounced in IFN-γ-treated cells compared to untreated ones (*p* ≤ 0.05) (Figures 2D,F).

Collectively, these findings suggested that *H. pylori*, by hindering the expression of CIITA in macrophages, affected the synthesis of HLA-II molecules, even in the presence of a strong inducer, such as IFN-γ. The decisive proof came from the quantification of the HLA-II synthetic rate in uninfected and infected macrophages, pre-treated or not with IFN-γ. Results shown in Figure 2G demonstrate that *H. pylori* inhibited the synthesis of HLA-II both in the absence and in the presence of the activating cytokine.

### *H. pylori* Infection Modulates the Expression of miRNAs Predicted to Target CIITA in Macrophages

**Figure 3:**
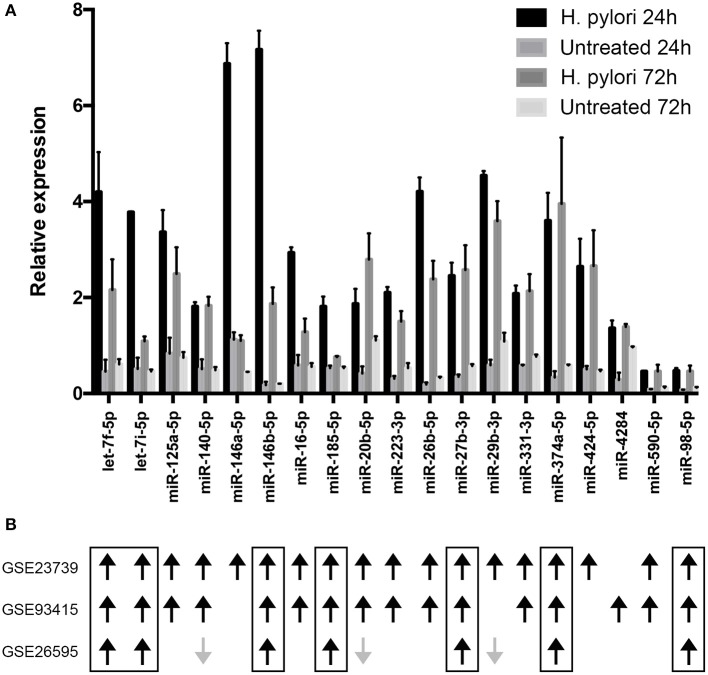
Relative expression of miRNAs putatively targeting CIITA transcript. **(A)** miRNA expression was evaluated by microarray in macrophages infected with *H. pylori* for 24 or 72 h. Data are expressed relative to the average of the expression of each miRNA. SD is relative to three biological replicates. Differentially expressed miRNAs were identified according to the significance analysis of microarray (SAM) using a median false discovery rate (FDR) of 0%. **(B)** Results of meta-analyses. Black arrows indicate miRNAs up-regulated in gastric cancer, while gray arrows indicate miRNAs down-regulated in gastric cancer. The Limma (Linear Models for Microarray Analysis) algorithm implemented in R package was used to identify differentially expressed miRNAs statistically significant. Benjamini and Hochberg correction was used to correct *p* values for multiple testing and it was considered if lower than 0.05.

To define mechanism(s) by which *H. pylori* antagonized the synthesis of CIITA, we considered the capacity of the bacterium to modulate miRNA expression in macrophages (12). We evidenced that the expression of 142 miRNAs was affected in macrophages by *H. pylori* infection (Supplementary Table 1 and Supplementary Figure 2). Among miRNAs up-regulated in both tested conditions (24 and 72 h of *H. pylori* infection), 19 were predicted to target CIITA (let-7f-5p, let-7i-5p, miR-125a-5p, -140-5p, -146a-5p, -146b-5p, -16-5p, -185-5p, -20b-5p, -223-3p, -26b-5p, -27b-3p, -29b-3p, -331-3p, -374a-5p, -424-5p, -4284, -590-5p, and -98-5p) (Figure 3A and Supplementary Table 2). Compelling evidence have demonstrated that miRNA expression is deregulated in human cancer and that this affects the hallmarks of cancer, including sustaining proliferative signaling, evading growth suppressors, resisting cell death, activating invasion and metastasis, and inducing angiogenesis (26, 27). Moreover, considering that *H. pylori* infection is the major risk factor associated with the development of GC (28, 29), and that a prevalence of M2-skewed tumor-infiltrating macrophages (TAM) expressing low HLA-II is associated with a poor prognosis (30), we prioritized miRNAs according to their deregulation in GC. Differentially expressed miRNAs in GC were identified using a meta-analysis approach basing on data published on GEO database (GEO ID: GSE26595, GSE23739, and GSE93415). By this method, we considered different techniques to profile miRNA expression and a total of 110 GC patients and 68 controls (Supplementary Table 3). The high number of deregulated miRNAs in GC reinforced the notion that miRNAs are crucial in the initiation and progression of tumors [Supplementary Table 4 and Supplementary Figure 3; (31)]. We also considered GSE78091 and GSE54397 gene sets, but we did not obtain any statistically significant differences among miRNAs expressed in normal and cancer samples. We evidenced that without distinguishing between the two GC histotypes (diffuse or intestinal) and among different cancer grades (GSE23739 and GSE93415 datasets), 15 miRNAs up-regulated in tumor samples from both datasets were also up-regulated in macrophages infected with *H. pylori* for 24 or 72 h (Figure 3B and Supplementary Figure 3). On the contrary, considering tumor histotype and grade (GSE26595), only 10 miRNAs up-regulated by *H. pylori* infection resulted modulated in both diffuse and intestinal GC. Among them, seven miRNAs were up-regulated in both infected macrophages and in GC (let-7f-5p, let-7i-5p, miR-146b-5p,−185-5p,−27b-3p,−374a-5p, and−98-5p), while three had an opposite behavior (miR-140-5p,−20b-5p,−29b-3p) (Figure 3B and Supplementary Figure 3). To assess the role of miRNAs induced by *H. pylori* in the regulation of CIITA, we considered all seven miRNAs with concordant expression in macrophages infected with *H. pylori* and in all datasets considered for meta-analyses.

### Let-7f-5p, Let-7i-5p, miR-146b-5p, and -185-5p Target CIITA mRNA

**Figure 4:**
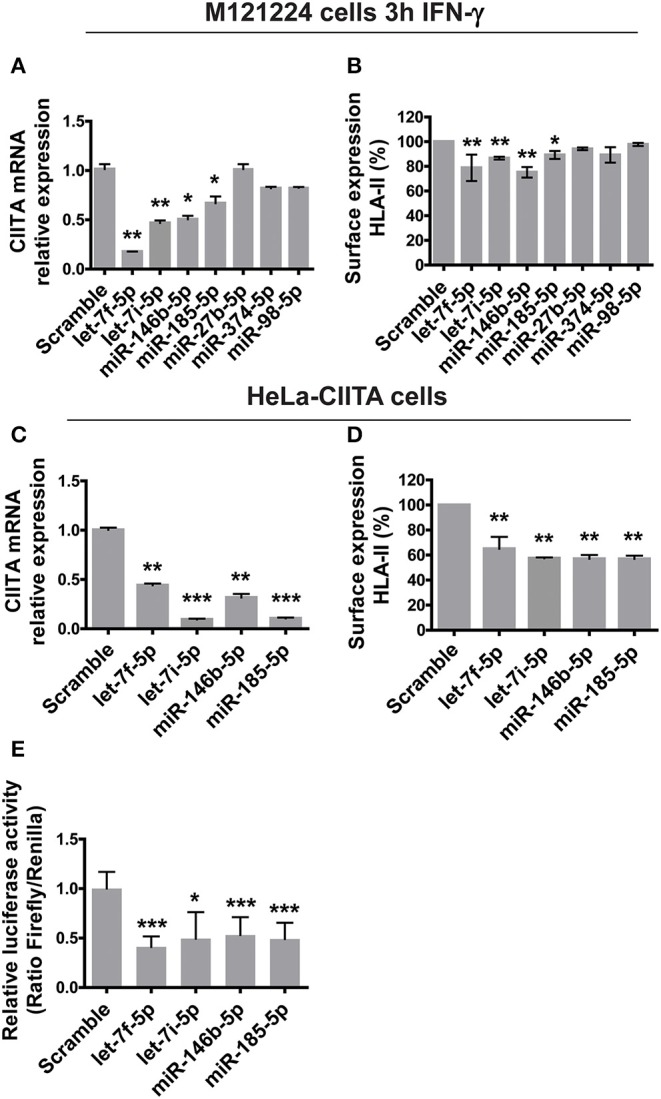
Functional analysis of miRNAs modulated by *H. pylori*. **(A)** Relative expression of mRNA encoding CIITA in M121224 melanoma cells activated for 3 h with 50 ng/ml IFN-γ and then transfected with pCMV-miR plasmid carrying the indicated miRNAs for 48 h. Data were normalized to an endogenous reference gene (β-actin). Values of cells expressing miRNA scramble were taken as reference and set as 1. **(B)** Surface expression of HLA-II in M121224 cells treated as in **(A)**. HLA-II expression in transfected cells was relative to control cells (expressing miRNA scramble). Data are expressed as mean ± SEM of three independent experiments, performed with 3 different cell preparations. **(C)** Relative expression of mRNA encoding CIITA in HeLa-CIITA cells transfected with pCMV-miR plasmid carrying the indicated miRNAs for 48 h. Data were normalized to an endogenous reference gene (β-actin). Values of cells expressing miRNA scramble were taken as reference and set as 1. **(D)** Surface expression of HLA-II in HeLa-CIITA cells treated as in **(C)**. HLA-II expression in transfected cells was relative to control cells. Data are expressed as mean ± SEM of three independent experiments, performed with three different cell preparations. **(E)** Luciferase assay. Luciferase assay supports the interaction between let-7f-5p,−7i-5p, miR-146b-5p, and -185-5p and the 3′-UTR of CIITA. As controls, we used cells transfected with pCMV-miR plasmid encoding miRNA scramble. Data are expressed as mean ± SEM of three independent experiments. Significance was determined by Student's *t*-test. *p < 0.05; **p < 0.01; and ***p < 0.001.

To validate CIITA as target of the seven considered miRNAs not only in macrophages but also in different cells, and considering the low transfection efficiency of macrophages, we adopted two different cell models: a melanoma cell line (M121224) stimulated with IFN-γ (24) and HeLa cells stably expressing CIITA (HeLa-CIITA) (32). M121224 were stimulated 3 h with IFN-γ before transfecting for 48 h with plasmids encoding single miRNAs. Let-7f-5p, let-7i-5p, miR-146b-5p, and -185-5p significantly down-regulated the expression of CIITA, while no changes in the expression of CIITA were detected in cells transfected with miR-27b-3p, -374a-5p, and -98-5p (Figure 4A). The pattern was recapitulated by the surface expression of HLA-II (Figure 4B). The four effective miRNAs were used to transfect HeLa-CIITA cells, and the above findings were confirmed, in terms of both CIITA mRNA expression and HLA-II expression (Figures 4C,D). The overexpression of the four miRNAs was confirmed by qRT-PCR in both M1212224 and HeLa-CIITA transfected cells (Supplementary Figure 4) and the interaction between let-7f-5p, let-7i-5p, miR-146b-5p, and -185-5p and CIITA 3′-UTR was substantiated by luciferase assay (Figure 4E).

**Figure 5:**
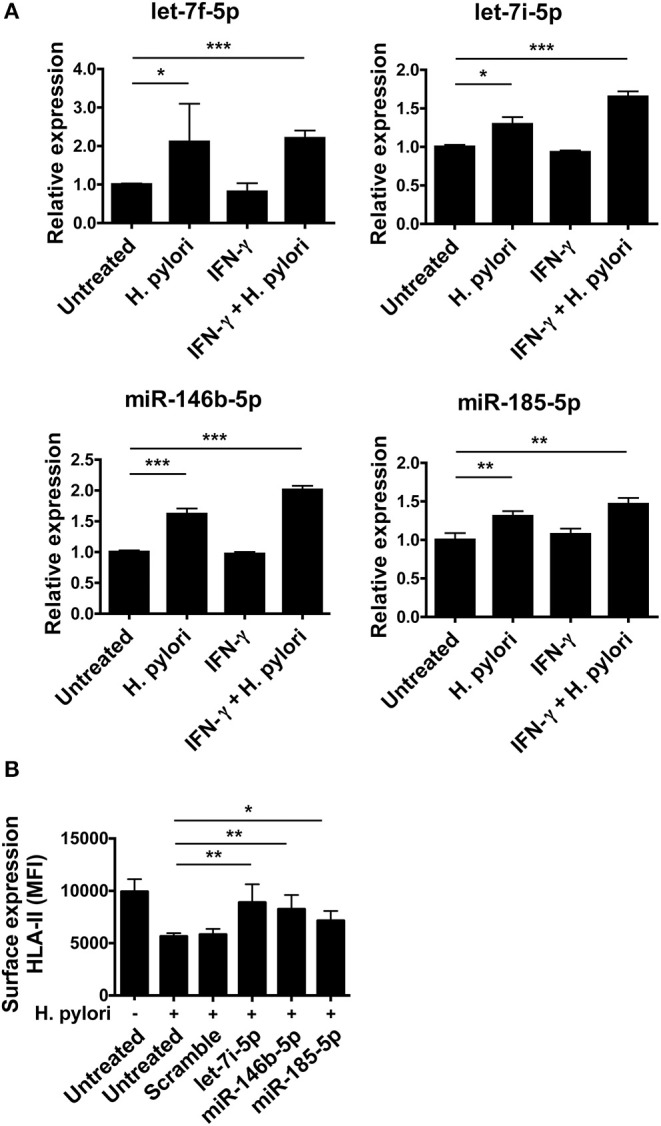
Modulation of miRNA expression in *H. pylori*-infected macrophages. **(A)** Relative expression of miRNAs in macrophages activated or not for 6 h with 20 ng/ml IFN-γ and then infected with *H. pylori* (MOI 10) for 48 h. Data were normalized to an endogenous reference gene (U6). Values of untreated cells were taken as reference and set as 1; the expression levels for treated cells were relative to the expression of untreated cells. **(B)** Surface expression of HLA-II in infected macrophages silenced for the indicated miRNAs. Data are expressed as mean ± SEM of three independent experiments, performed with cells from three different donors. Significance was determined by Student's *t*-test. *p < 0.05; **p < 0.01; and ***p < 0.001.

**Figure 6:**
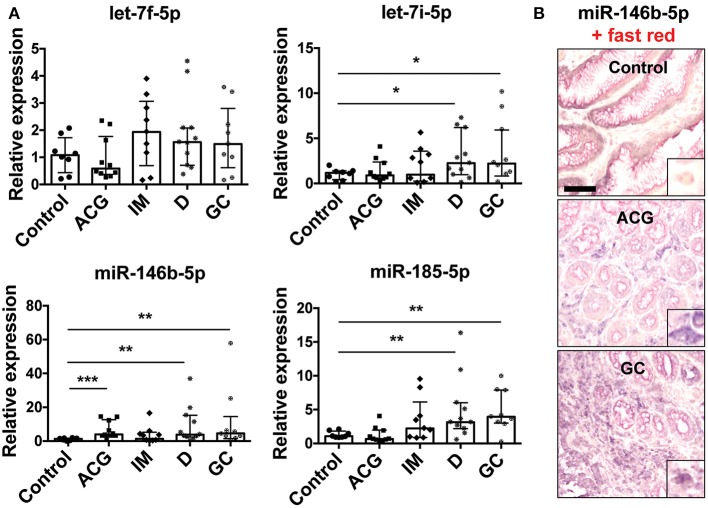
Modulation of miRNA expression in gastric biopsies. **(A)** Relative expression of miRNAs in gastric biopsies from control biopsies (control, *n* = 8), patients with active non-atrophic chronic gastritis (ACG, *n* = 10), patients with intestinal metaplasia (IM, *n* = 9), with dysplasia (D, *n* = 11), and patients with gastric adenocarcinoma (GC, *n* = 9). Data were normalized to an endogenous reference gene (U6). Values of control biopsies were taken as reference and set as 1; the expression levels for patients were relative to the expression of control biopsies. Data are expressed as median with interquartile range (IQR). Significance was determined by the Mann–Whitney *U*-test. *p < 0.05; **p < 0.01; and ***p < 0.001. **(B)** Representative images of miR-146b-5p ISH in control biopsies, ACG and GC. Scale bar: 100 μm. In each case, a higher magnification (10×) of miR-146b-5p expression in macrophages is shown as right bottom inlet.

qRT-PCR on macrophages infected with *H. pylori* confirmed the up-regulation of let-7f-5p, let-7i-5p, miR-146b-5p, and -185-5p, evidenced by the microarray analysis. The treatment of macrophages with IFN-γ that stimulates CIITA transcription did not affect the expression of any of these miRNAs. Interestingly, *H. pylori* infection induced the up-regulation of all four miRNAs in macrophages treated with IFN-γ similarly to those non-treated with IFN-γ or even more (Figure 5A). Therefore, IFN-γ does not affect the ability of *H. pylori* to stimulate the expression of miRNAs regulating CIITA in macrophages. Notably, the expression of HLA-II in infected macrophages was rescued by inhibiting the action of let-7i-5p, miR-146b-5p, and miR-185-5p (Figure 5B). Let-7f-5p did not display differential expression pattern between normal mucosa and *H. pylori*-associated diseases (Figure 6); thus, we excluded it from miRNA inhibition experiments.

### MiRNAs Targeting the CIITA mRNA Are Up-Regulated in GC

To confirm the results obtained by meta-analysis approach on miRNA expression in GC (110 patients with GC and 68 controls) and to investigate whether our *in vitro* data have an *in vivo* correlation, we analyzed the expression of let-7f-5p, let-7i-5p, miR-146b-5p, and -185-5p in tissues from patients with a histological and serological established diagnosis of *H. pylori* infection. We considered 10 patients with active non-atrophic chronic gastritis, 9 patients with intestinal metaplasia, 11 with dysplasia (low and high grade), and 9 patients with gastric adenocarcinoma, thus including all the lesions that define the carcinogenetic process of the intestinal variant of GC (i.e., the Correa's cascade). The expression of let-7i-5p, miR-146b-5p, and -185-5p was increased in GC samples and the early neoplastic condition (dysplasia), confirming results obtained by meta-analysis approach and in accordance to several works demonstrating the importance of these miRNAs in different tumors [Figure 6A; (33–38)]. Despite meta-analysis results that revealed that let-7f-5p increased, we did not confirm such an alteration in our samples. Let-7f-5p has been found silenced in metastatic GC in association with an increased cell migration and invasion (39). The apparent discrepancy between our evidence and that published could be explained by the fact that all considered cancers in our series are of early stage. Among the three up-regulated miRNAs, miR-146b-5p was the most up-regulated in our patients and we revealed that it is mainly expressed by macrophages and, to a less extent, by plasma cells and epithelial cells (Figure 6B).

## Discussion

Throughout the long-lasting co-existence between *H. pylori* and its human host, the bacterium has evolved a plethora of mechanisms to actively elude innate and adaptive immune response, which, collectively, account for the infection persistence (7). In the absence of antibiotic treatment, the latter is responsible for the maintenance of a status of chronic inflammation that can evolve in serious gastric diseases, including GC (1). The gastric mucosa of *H. pylori*-infected people has an increase in activated macrophages that produce several cytokines, including IL-12, responsible for inflammation and initiating Th1-type responses (5, 40). The role of Th1 cells in activating the killing potential of macrophages, through the release of IFN-γ, is established; however, the evidence that, in spite of the presence of these effector cells, *H. pylori* successfully establishes a persistent infection suggests that Th1 cells might be ineffective to clear the pathogen.

Mechanisms responsible for this failure are partially understood and involve the manipulation and inhibition of effector T cell responses (7). However, the ability of *H. pylori* in disrupting the process of phagosome maturation that leads to formation of a hybrid phagosome–endosome–lysosome compartment, with reduced degradative capacity (9), may also exert a profound influence on the persistence of bacteria. Indeed, an efficient bacterial degradation and a successful antigen presentation are essential for macrophages to be recognized and activated by Th1 lymphocytes. In the same line of evidence, we revealed that macrophages infected by *H. pylori* have an impaired capacity to present antigens and to activate the T cell-mediated immunity. This relies on a mechanism involving the immune receptor CD300e, but it reflects also a direct action of the bacterium on phagocytes (12).

Many bacteria evolved strategies for inhibiting HLA-II expression to evade immune recognition (41). Mycobacteria, for example, subvert antigen presentation by macrophages by inducing specific isoforms of the transcription factor C/EBP that suppress transcription of CIITA (42). In this study, we describe the down-regulation of HLA-II in macrophages, based on the post-transcriptional modulation of CIITA by miRNAs. The role of these short RNA sequences as regulators that help the fine-tuning of the immune response is substantiated by experimental evidence (43) and the participation of miRNAs in host immune defense against bacterial infections, including those caused by *H. pylori*, has also been demonstrated (44).

Here, we show that *H. pylori* triggers the up-regulation of four miRNAs (let-7f-5p, let-7i-5p, miR-146b-5p, and -185-5p) that modulate the expression of CIITA and therefore that of HLA-II, both at the mRNA and protein level. To date, only miR-145 and -198 have been shown to regulate the expression of CIITA, and only for miR-145 was the ability to attenuate the induction of CIITA by IFN-γ demonstrated (45).

Considering that Th1 cells secreting IFN-γ predominate in the gastric mucosa of *H. pylori*-infected patients, the finding that *H. pylori* affected the capacity of the cytokine to elicit the up-regulation of CIITA, keeping the amount of HLA-II on the cell surface at a basal level, was found to be interesting. The effect was observed both in macrophages pre-activated by IFN-γ and in those activated after the infection, even if in the latter case the intracellular signaling elicited by IFN-γ was probably prevented also because of the depletion of cholesterol from the lipid rafts, by the bacterial cholesterol-α-glucosyltransferase [Supplementary Figure 5; (46)].

Overall, our data corroborate the notion that the bacterium impairs the antigen presentation capacity of non-activated macrophages (12) but, most importantly, it hinders the activation of them by IFN-γ. Moreover, we verified that the impact of *H. pylori* on the HLA-II expression is strain independent and does not require the integrity of the pathogenicity island CagPAI, the production of VacA or HP-NAP, and full motility (Supplementary Figure 6).

The inability of macrophages in degrading bacteria efficiently and the failure in antigen presentation could act synergistically to render macrophages a protective niche for the bacterium and could represent one of the mechanisms by which the bacterium survives in the human gastric mucosa, despite a robust Th1 immune response.

**Figure 7:**
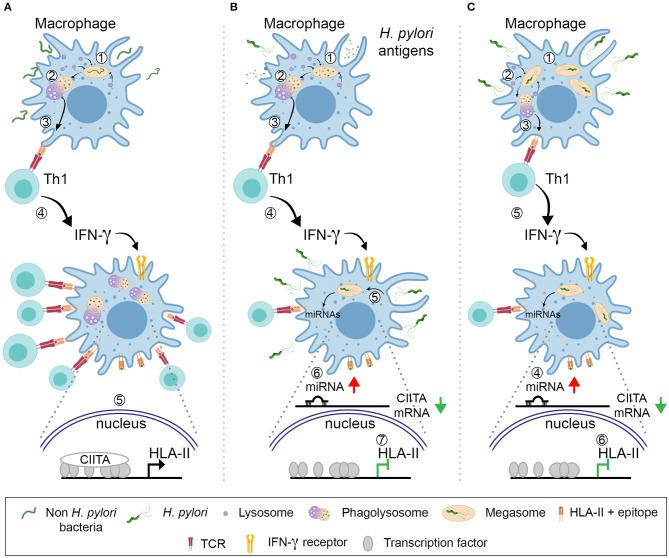
Model for the *H. pylori* action against the antigen presentation by HLA-II. **(A)** A common bacterium is engulfed by macrophages (1). Upon killing and digestion in the phagolysosomes (2), bacterial antigens are displayed by HLA-II molecules on the plasma membrane (3). Specific Th1 cells recognize the peptide–HLA-II complexes by TCR and release IFN-γ (4), which activates the transcription of CIITA (5). The increased number of HLA-II-epitopes that can be exposed on the cell surface maximizes the number of T cells activated and amplify the intensity of the ensuing immune response. **(B)** Macrophages internalize *H. pylori* antigens released by bacteria (1). Antigens are processed in phagolysosomes (2) and presented in HLA-II to Th1 lymphocytes (3). The latter release IFN-γ (4), but the cytokine action is hindered because of the surrounding/engulfed bacteria (5) that up-regulate the expression of CIITA-targeting miRNAs in macrophages (6). The number of HLA-II molecules does not increase (7) and the activation of Th1 cells with different epitope-specificity is compromised. **(C)** *H. pylori* infect macrophages and are phagocytosed (1); bacteria accumulate in megasomes and partial digestion occurs (2); bacterial epitopes are presented in HLA-II to Th1 lymphocytes (3). Concomitantly, the up-regulation of CIITA-targeting miRNAs is elicited (4) and this nullifies the effect of IFN-γ (5), in terms of HLA-II synthesis (6).

Based on our results, we propose the following scenario: *H. pylori* infiltrates the gastric mucosa where it is engulfed by dendritic cells that, despite an altered activation caused by several virulence factors (47), elicit an adaptive immune response, with a strong prevalence of Th1 cells. Meanwhile, macrophages are also exposed to *H. pylori* or bacterial products derived from dying bacteria. For macrophages that are engaged in immunological synapses with Th1 lymphocytes and are presenting bacterial soluble antigens, engulfed from the extracellular medium, one can envisage that the effect of IFN-γ on HLA-II expression might be hindered because of surrounding/engulfed bacteria. They could trigger a signal culminating in the up-regulation of CIITA-targeting miRNAs by acting either at the plasma membrane or upon phagocytosis; discriminating between these two potential mechanisms will require further investigation. On the other hand, macrophages exposed to bacteria, or engulfing them before receiving the stimulation of IFN-γ, might be even more affected because of both the interference with the cytokine signaling due to the cholesterol depletion and the impaired transcription of CIITA (Figure 7).

It is worthy to note that three of four miRNAs up-regulated in infected macrophages (let-7i-5p, miR-146b-5p, and -185-5p) are essential for HLA-II modulation. The same miRNAs are increased in GC samples and in the early neoplastic condition, confirming results obtained by meta-analysis approach and in accordance to several works demonstrating the importance of these miRNAs in different tumors (31). MiR-185-5p, in particular, has been recognized as relevant for tumor initiation and progression in several cancers, including GC, and it is a promising prognostic biomarker to predict survival and relapse of GC patients (48). MiR-146b-5p, identified also in gastroduodenal ulcers associated with *H. pylori*, is the most up-regulated miRNA in GC samples (49) and here we showed that it is mainly expressed by macrophages. However, since at least seven predominant TAM populations characterized by specific combinations of markers on individual cells accumulate in GC (50), proving that the population of macrophages that expresses miR-146b-5p weakly expresses HLA-II, it is mandatory to support the notion that a causative relationship exists *in vivo* between the overexpression of miR-146b-5p and the down-regulation of the antigen-presenting molecules in TAMs. These findings, together with the definition of the spatial distribution of miR-146b-5p^+^/HLA-II^low^ TAMs in the different tumor regions (core, edge, or margin) would contribute to the advancement of the knowledge of the immune environment of GC.

## Materials and Methods

### Ethics Statement

Investigation has been conducted following the ethical standards, the Declaration of Helsinki, and national and international guidelines and has also been approved by the authors' institutional review board (4406/AO/18, Comitato Etico per la Sperimentazione Clinica a servizio delle Aziende Sanitarie della Provincia di Padova, Padova, Italy).

Peripheral blood mononuclear cells utilized in this study derived from buffy coats obtained from healthy blood donors, as anonymously provided by the Transfusion Centre of the Hospital of Padua. Written informed consent for the use of buffy coats for research purposes was obtained from blood donors by the Transfusion Centre. Data related to human samples were all analyzed anonymously. Human leukocytes, provided by the Transfusion Centre of the Hospital of Padua, were obtained not consequently to experimentation on human beings but as a consequence of voluntary and informed blood donation for transfusions: no approval of the Ethics Committee is needed in such cases in our institution.

### Cell Cultures

#### Monocyte Isolation, Macrophage Differentiation, and Cell Treatment

Monocytes derived from buffy coats were prepared as described previously (51). For macrophage differentiation, 5 × 10^5^ monocytes, seeded in 24-well plates, were cultured in RPMI 1640 medium (Euroclone), 20% FBS, 4 mM HEPES (Gibco, ThermoFisher Scientific), and 50 μg/ml gentamycin (Gibco, ThermoFisher Scientific) in the presence of 100 ng/ml M-CSF (Miltenyi Biotec) for a 6-day differentiation. Cells were infected with bacteria (5 × 10^6^ CFU/ml, MOI = 10) in RPMI 1640 20% FBS with no M-CSF and no antibiotic. Medium was not replaced during the whole duration of the infection. Biological triplicates were performed with cells from three buffy coats handled on the same day.

For evaluating the impact of *H. pylori* on IFN-γ-induced signaling, on one hand, cells were activated with 20 ng/ml IFN-γ for 6 h, washed with RPMI 20% FBS, and infected for further 48 h in culture medium without antibiotic. On the other hand, cells were infected 16 h with *H. pylori*, washed with RPMI 20% RPMI, and further incubated with IFN-γ for 48 h.

#### M121224 and HeLa-CIITA Cell Culture and Cell Treatment

Melanoma cell line M121224 (52) was cultured in RPMI 1640 medium, 10% FBS, 10 mM HEPES, 100 U/ml penicillin, and 100 μg/ml streptomycin antibiotics (Sigma, Germany).

HeLa cells stably expressing CIITA (HeLa-CIITA, kindly provided by Prof. P. Cresswell, New Haven CT, USA) were grown in DMEM medium (Euroclone), 10% FBS, 10 mM HEPES, and penicillin–streptomycin antibiotics (Sigma, Germany).

M121224 were pre-treated for 3 h with 50 ng/ml IFN-γ and both M121224 and HeLa CIITA cells were transfected with specific or scramble miRNAs encoding plasmids. After 48 h, CIITA mRNA expression was evaluated by qRT-PCR and HLA-II surface expression was evaluated by FACS analysis.

#### LNA Transfection and Infection of Macrophages

To silence let-7i-5p, miR-146b-5p, and -185-5p, monocyte-derived macrophages were transfected with 25 nM of LNA complementary to let7i-5p (ID: YI04101178), miR-146b-5p (ID: YI04101999), and -185-5p (ID: YI04100512) or with scramble LNA (ID: YI00199006), provided by Qiagen. Cell transfection was performed with the Viromer Green transfection system (Lipocalyx) following the manufacturer's instructions. After 24 h, cells were washed with RPMI 20% FBS and infected with *H. pylori* (MOI = 10) for further 48 h in culture medium without antibiotic.

### Bacteria

The following *H. pylori* strains were used: CagA^+^/VacA^+^ strain P12 (53), kindly provided by Prof. W. Fischer (Munich, Germany); CagA^+^/VacA^+^ strain 342, CagA^+^/VacA^+^ strain G27, and their isogenic mutants Δhp-nap and ΔcagPAI (54, 55), kindly provided by A. Covacci (Novartis Vaccines, Siena, Italy); Cag^+^/VacA^+^ strain SPM326 and its isogenic mutant ΔvacA (55, 56), kindly provided by J. L. Telford (Novartis Vaccines); strain N6 and its isogenic mutant ΔflaA (57), kindly provided by Prof C. Josenhans (Hannover, Germany).

Bacteria were maintained in 5% CO_2_ at 37°C on Columbia agar plates supplemented with 5% horse blood (Oxoid) and antibiotics (trimethoprim 10 μg/ml, cefsulodin 6 μg/ml, vancomycin 5 μg/ml, and amphotericin B 8 μg/ml) (Sigma, Germany). For mutant strains, kanamycin 20 μg/ml was added to the above mixture. Colonies were taken directly from plates and resuspended in RPMI 20% FBS without any antibiotic. Bacterial count was performed by determining the optical density (OD) at 600 nm (1 OD = 10^9^ CFU/ml). Before proceeding with the infection experiments, bacteria motility was verified at the optical microscope. Handling of biohazardous material was performed in a dedicated bio-safety level 2 room and approved by the local prevention and hygiene security committee.

### Patients

The cases considered in this study were retrospectively collected from the Surgical Pathology and Cytopathology Unit at the University of Padua (January 2010 to December 2018). All patients were Caucasian and underwent endoscopy/surgery at Padua University Hospital and all the samples were formalin-fixed and paraffin-embedded (FFPE).

The study was conducted on a total of 18 antral mucosa endoscopic biopsy samples obtained from 8 dyspeptic patients (with no significant histological alterations and used as control) and from 10 *H. pylori*-positive non-atrophic active chronic gastritis patients (biopsies were obtained before *H. pylori* eradication therapy). A further series of 29 samples were selected and microdissected from 10 patients surgically treated for *H. pylori*-positive early-stage intestinal-type GCs (all G1/G2 and all pT1 or pT2). In particular, 9 samples of extensive intestinal metaplasia (≥85%), 11 samples of dysplasia [both low-grade and high-grade lesions were considered and defined as for WHO classification (58)], and 9 samples of adenocarcinoma were considered. All 29 samples presented moderate chronic inflammatory infiltration with moderate/severe *H. pylori*-related activity, as assessed according to the Sydney system (59). Patients had a median age of 63.3 years (range 45–79 years) and were 9 males and 17 females.

In the *H. pylori*-positive samples, the bacterium was assessed by histology (modified Giemsa staining) and confirmed by clinical history, rapid urease testing, and/or ELISA (*H. pylori*-specific IgG Abs; GastroPanel, Biohit HealthCare) (12).

### Flow Cytometry

Cells were harvested from culture plates using 5 mM Na–EDTA in PBS, pH 7.5, and incubated for 15 min at RT with 10% human serum to saturate Fc receptors. 5 × 10^5^ cells were stained with a monoclonal antibody anti-HLA-II (clone L243, Ebiosciences). We used fixable cell viability dye eFluor780 (Ebiosciences) to exclude dead cells from the analysis. The latter were <5–10% of the total cell number, regardless of the treatment. Cells were resuspended in FACS buffer (PBS, 1% BSA) and analyzed by FACSCanto II (Becton Dickinson). Forward and side scatter light were used to identify cell populations. Values were expressed as the median fluorescence intensity (MFI) of live cells positive for HLA-II. All data were analyzed using FlowJo software, version 10.3 (Tree Star Inc.).

### Western Blot

Macrophages were lysed in RIPA buffer, and proteins were quantified by BCA protein assay kit (ThermoFisher Scientific), according to the manufacturer's instructions. Equal amounts of proteins were resuspended in NuPAGE LDS sample buffer (Novex, Life Technologies) supplemented with 50 mM DTT and denaturated for 5 min at 100°C. Samples were separated electrophoretically in NuPAGE Bis-Tris 4–12% polyacrylamide gel (Novex, Life Technologies), and proteins were subsequently transferred onto PVDF membranes (Amersham). Membranes were blocked with 3% non-fat milk in Tris-buffered saline (TBS, 50 mM Tris–HCl, pH 7.6, 150 mM NaCl) containing 0.1% Tween20® (Sigma-Aldrich), and antigens were revealed using a mouse anti-human HLA-II α-chain monoclonal antibody (1:3,000, clone TAL-1B5 Abcam) and a rabbit anti-human vinculin antibody (1:5,000, Origene). Blots were washed three times with TBS plus 0.1% Tween20® and incubated for 1 h at RT with horseradish peroxidase-conjugated anti-mouse IgG (Novex, Life Technologies) or anti-rabbit IgG (Millipore) secondary antibody, respectively. Blots were developed with enhanced chemiluminescence substrate (EuroClone), and protein bands were detected using ImageQuant™ LAS 4000 (GE Healthcare Life Science).

### HLA-II Synthetic Rate

Macrophages were incubated 1 h in methionine-free medium to deplete methionine reserves. Cells were labeled with the methionine analog L-azidohomoalanine 50 μM (AHA, Click-IT^TM^ Invitrogen) for 2 h and lysed in 1% SDS 50 mM Tris–HCl. All newly synthetized proteins were tagged with biotin alkyne (Click-IT™ Invitrogen) according to the manufacturer's protocol. Proteins were pulled down with NeutrAvidin agarose resin (Pierce), processed for Western blot, and developed for HLA-II.

### RNA Extraction

#### Macrophages

Total RNA was extracted with TRIzol reagent (Thermo Fisher Scientific) according to the manufacturer's protocol. RNA was quantized using NanoDrop 1000 spectrophotometer (Nanodrop). RNA integrity and content of miRNAs in each sample were assessed by capillary electrophoresis using the Agilent Bioanalyzer 2100 with the RNA 6000 Nano and the Small RNA Nano LabChips, respectively (Agilent Technologies). Only samples with an RNA integrity number >7 and with a concentration of small RNAs <30% were used for miRNA or mRNA gene expression analysis.

#### Biopsies

miRNA extraction from formalin-fixed, paraffin-embedded (FFPE) tissue sections was performed using the miRNeasy FFPE Kit (Qiagen) according to the manufacturer's protocol. Dysplastic/cancer samples were manually microdissected to ensure that each sample contained at least 90% of lesioned mucosa. The percentage of the target lesion as obtained by manual microdissection was further validated on an adjunctive H&E histology section. The lowest RNA integrity number accepted for further analyses was 2.5.

### miRNA Microarray Expression Profiling and Data Analysis

Microarray experiments were performed using Agilent Human miRNA Microarray 8 × 60 K platform (Agilent Technologies) based on miRbase V.19. Two hundred nanograms of total RNA was labeled using miRNA Complete Labeling and Hyb Kit (Agilent Technologies), according to the manufacturer's protocol. Labeled RNA was hybridized onto microarray slides using a rotational oven at 55°C for 22 h. After hybridization, microarray slides were washed and scanned in an Agilent microarray scanner (model G2565CA). Agilent Feature Extraction software version 10.5.1.1 was used for image analysis. Microarray data expression is available in the U.S. National Center for Biotechnology Information Gene Expression Omnibus (GEO) database (GSE98641).

Raw microarray data were filtered for the number of miRNAs presenting an expression value above background (allowed 50% of undetected values for each miRNA) and then normalized according to loess cyclic algorithm (60). Differentially expressed miRNAs were identified according to the significance analysis of microarray (SAM) (61), using a median false discovery rate (FDR) of 0% (Supplementary Table 1). miRNA targets were recovered by different algorithms using default parameters: Microt4 (62), miRanda (63), mirbridge (64), miRDB (65), miRMap (66), miRNAMap (67), Pictar2 (68), PITA (69), RNA22 (70), RNAhybrid (71), Targetscan (72), and miRWalk (73). Results are listed in Supplementary Table 2.

Data for differentially expressed miRNAs targeting CIITA were extracted from microarray experiments and represented referring to the average expression of each miRNA in all samples.

### Meta-Analysis

Five different datasets describing miRNA expression in GC and control samples were downloaded from Gene Expression Omnibus database (GSE26595, GSE23739, GSE93415, GSE78091, and GSE54397). Processed data were used to identify differentially expressed miRNAs. GSE26595 data were quantile normalized (74), GSE23739 by median centering across probes (75), GSE93415 by Lowess (Locally Weighted Scatterplot Smoothing) algorithm, GSE78091 by median normalization, while GSE54397 were not processed for inter-array normalization (76). The limma (Linear Models for Microarray Analysis) algorithm (77) implemented in R package was used to identify differentially expressed miRNAs statistically significant. Benjamini and Hochberg correction was used to correct *p*-values for multiple testing and corrected *p*-value lower than 0.05 was considered significant to identify the list of differentially expressed miRNAs. The only dataset containing information about tumor type and grade was the GSE26595. Samples in this dataset were compared as follows: controls vs. diffuse GC grades 1 and 2, controls vs. diffuse GC grades 3 and 4, controls vs. diffuse GC all grades, controls vs. intestinal grades 1 and 2, controls vs. intestinal GC grades 3 and 4, controls vs. intestinal GC all grades. In other datasets, comparisons performed were controls vs. GC samples. Lists of differentially expressed miRNAs from each comparison were compared through Venn diagrams [Supplementary Figure 2; (78)].

### qRT-PCR

#### mRNAs

One microgram of total RNA was retrotranscribed using the Superscript II enzyme (Thermo Fisher Scientific) and oligod(T) as starting primer. Retrotranscription was performed at 42°C for 2 h. After precipitation, 5 ng of cDNA was used in qRT-PCR reaction performed in a 7900HT Fast Real-Time PCR System (Thermo Fisher Scientific). qRT- PCR reaction was performed in 10 μl using SYBR Green master mix (Thermo Fisher Scientific), according to the following cycle: 95°C for 5 min; 95°C for 15 s, 60°C for 1 min, for 40 cycles. Experiments were performed with at least three biological and technical replicates. For each sample, data were normalized to the endogenous reference gene β-actin and confirmed using a different reference gene (18S; data not shown). Primer sequences were reported in the Supplementary Table 5.

#### miRNAs

miRNA expression was performed using TaqMan® MicroRNA Assays (Thermo Fisher Scientific). Retrotranscription of each specific miRNA was accomplished using TaqMan® MicroRNA Reverse Transcription Kit with 10 ng of total RNA (Thermo Fisher Scientific) following the manufacturer's instructions. RT-PCR was then performed using TaqMan® Universal PCR Master Mix II, no UNG (Thermo Fisher Scientific) and the specific primers from TaqMan® MicroRNA Assay. At least three replicates were performed for each reaction. miRNAs expression was normalized to the U6 small nuclear RNA (U6 snRNA) (Thermo Fisher Scientific—Assay ID 001973). miRNAs analyzed were let-7f (Thermo Fisher Scientific—Assay ID 000382), let-7i (Thermo Fisher Scientific—Assay ID 002221), miR-146b (Thermo Fisher Scientific—Assay ID 001097), and miR-185 (Thermo Fisher Scientific—Assay ID 002271). Reactions were run in 96 CFX (BioRad) with the following program: 95°C for 10 min; 35 cycles of 95°C for 15 s; and 60°C for 1 min.

Data analysis was carried on according to ΔΔCt method for both mRNAs and miRNAs.

### miRNA Overexpression

To overexpress miRNAs in M121224 and HeLa-CIITA cells, pCMV-MIR vectors were used (Origene): human MIRLET7i (ID: MI0000434), human MIRLET7f (ID: MI0000067), human MIR185 (ID: MI0000482), human MIR146b (ID: MI0003129), human MIR27b (ID: MI0000440), human MIR98 (ID: MI0000100), and human 374a (ID: MI0000782). Cells transfected with a scrambled plasmid (ID: PCMVMIR) served as control. Cell transfections were performed for 48 h with the TransIT-X2® transfection system (Mirus) with 500 ng of the specific vector.

### Luciferase Assay

M121224 cells were transfected with 1.5 ng/μl PCMV-MIR vector expressing specific or scramble miRNA and 100 pg/μl of pmirGLO Dual-Luciferase miRNA Target Expression Vector (Promega), containing the target sequence (Supplementary Table 6). pCMV-MIR vector encoding miRNA scramble was used as control. Assays were performed using Dual-Luciferase Reporter Assay (Promega) and measuring firefly and Renilla luciferase activities in a Turner Designs TD-20/20 Luminometer (DLReadyTM, Promega). miRNA transfections were replicated independently at least three times.

### miR-146b-5p *in situ* Hybridization (ISH) and Immunohistochemical Analyses

Original slides (4–6 μm thick) obtained from the paraffin-embedded tissue samples were probed with 5′-biotinilated LNA matching miR-146b-5p (Exiqon). Tissue sections were digested with ISH protease 1 (Ventana Medical Systems) and ISH was performed as previously described, with minor modifications (79). Only cytoplasmic miR intensity was retained for evaluation. Five samples per condition (dyspeptic control, ACG and GC) were analyzed.

### Statistic

Data are reported as the mean ± SD or mean ± SEM. The Student's *t*-test and Mann–Whitney *U*-test were used for statistical analysis of the differences between experimental groups. *p* ≤ 0.05 were considered significant.

## Data Availability Statement

The datasets generated for this study can be found in the GSE98641, GSE26595, GSE23739, GSE93415, GSE78091, and GSE54397.

## Ethics Statement

The studies involving human participants were reviewed and approved by Investigation has been conducted in accordance with the ethical standards, the Declaration of Helsinki, and national and international guidelines and has also been approved by the authors' institutional review board (4406/AO/18). The patients/participants provided their written informed consent to participate in this study.

## Author Contributions

MB conceived the study and wrote the manuscript. SCa analyzed microarray data, conceived the study, and wrote the manuscript. GC differentiated and infected macrophages with *H. pylori*, performed flow cytometry analysis, and performed miRNA qRT-PCR analysis on macrophages and patients. MT performed Western blot and mRNA qRT-PCR analysis on *H. pylori*-infected macrophages. SCo performed protein synthesis assay. GS performed luciferase assay. GB performed experiments on macrophages activated with IFN-γ. FC performed the transfections of M121224 and HeLa-CIITA cells with plasmids encoding miRNAs. MF collected and analyzed patients. GM performed mRNA extraction from patients' samples.

### Conflict of Interest

The authors declare that the research was conducted in the absence of any commercial or financial relationships that could be construed as a potential conflict of interest.
