# Supplementary material for: Helicobacter pylori Dampens HLA-II Expression on Macrophages via the Up-Regulation of miRNAs Targeting CIITA
Source: Front Immunol. 2020 Jan 8;10:2923. doi: 10.3389/fimmu.2019.02923 (PMC6960189; doi:10.3389/fimmu.2019.02923)
Supplement: Supplementary file 3 [file Table_3.pdf]

Table S3. Gene sets and patient description. ID column represents the GEO accession used to recover data; technique column describes the technique used to profile miRNAs; patients column describes information about patients, and publication describes if data were described in a publication.

ID	Technique	Patients	Publication
GSE26595	Illumina Human v2 MicroRNA expression beadchip	- 50 primary gastric cancer tissues (27 samples: diffuse gastric cancer; 23 samples: intestinal gastric cancer).<br>Diffuse gastric cancer samples: 3 stage 1b, 4 stage 2, 6 stage 3a, 6 stage 3b, 8 stage 4.<br>Intestinal gastric cancer samples: 2 stage 1a, 6 stage 1b, 3 stage 2, 7 stage 3a, 2 stage 3b, 3 stage 4.<br>- 8 surrounding non-cancer tissues	[1]
GSE23739	Agilent-019118 Human miRNA Microarray 2.0 G4470B	- 40 gastric tumor tissue<br>- 40 gastric normal tissue	[2]
GSE93415	Exiqon miRCURY LNA microRNA array; 7th generation (miRBase 19.0)	- 20 primary tumor<br>- 20 healthy gastric mucosa	

REFERENCES

1. Lim, J. Y. *et al.* Overexpression of miR-196b and HOXA10 characterize a poor-prognosis gastric cancer subtype. *World J. Gastroenterol.* (2013). doi:10.3748/wjg.v19.i41.7078
2. Oh, H.-K. *et al.* Genomic Loss of miR-486 Regulates Tumor Progression and the OLFM4 Antiapoptotic Factor in Gastric Cancer. *Clin. Cancer Res.* **17**, 2657–2667 (2011).
