# Supplementary material for: Helicobacter pylori Dampens HLA-II Expression on Macrophages via the Up-Regulation of miRNAs Targeting CIITA
Source: Front Immunol. 2020 Jan 8;10:2923. doi: 10.3389/fimmu.2019.02923 (PMC6960189; doi:10.3389/fimmu.2019.02923)
Supplement: Supplementary file 5 [file Table_5.pdf]

Table S5. Sequence of primers used for qRT-PCR.

**Name**	**Sequence 5'-3'**
CIITA For	GGTCCAGGGTTTGAGTTCAT
CIITA Rev	TGATTTGGGGTGGCTTGTTA
β-actin For	TGAGATGCGTTGTTACAGGA
β-actin Rev	ACGAAAGCAATGCTATCA
18S For	CGGCTACCACATCCAAGGAA
18S Rev	GCTGGAATTACCGCGGCT
