# Supplementary material for: Helicobacter pylori Dampens HLA-II Expression on Macrophages via the Up-Regulation of miRNAs Targeting CIITA
Source: Front Immunol. 2020 Jan 8;10:2923. doi: 10.3389/fimmu.2019.02923 (PMC6960189; doi:10.3389/fimmu.2019.02923)
Supplement: Supplementary file 6 [file Table_6.pdf]

Table S6. Cloned sequence in pmirGLO vector.

Name	Sequence 5’-3’
pmirGLO-CIITA	ACCAAGGGGAATGTTTGCCTCTGCCTCTGACACACAGCTCTGTCCTGGGAGTGAGCTGGTTTTTAGCGGAGACGGAGTCCCACCTTGGCTGCAGGGAGTCCGAAAGGGACTTGGAAGCTCCGCTTTCTACCCAGCGAGCTGCCGGCACGACTGTTGCTTTTCACTCGGGCATAGTCTGCTCAGAAGCCCCAATTCAAGACATCTTAGCTTACTCCTGGTGGCAGTGGGAGCTGCCTGTTCAGCTCCAGCTCACCAGCCCCAGTGCCCACAGGATCAGTCTGATTCCCAAGCTCTGCTCCTCTCCCCAGCAAGTGAGAGCTGGGTGTCAAGAGGGTCTGAGGAATCCAGACCCGGCTGCGTGGTTGATGATCTTTGCAGACAGGCAAGCACCTCCTGCCTCGAACTGGCTGCAAAGGGTATCAGGTGCTGCCATCCAGGTTCAGCTTGTAAATAGCTCAGGTGTCACCCTGCAAGGGATCCCTGCACTTGCAGACTCTACAGAGGCCATGGGCCTCCTGTGTGTGCGCCTACAGGAGTGAGCACTAAGGTGTGCTCTGATCATCCACTGTGCCATGTGCCAGGTTTTT
