# Supplementary material for: Helicobacter pylori Dampens HLA-II Expression on Macrophages via the Up-Regulation of miRNAs Targeting CIITA
Source: Front Immunol. 2020 Jan 8;10:2923. doi: 10.3389/fimmu.2019.02923 (PMC6960189; doi:10.3389/fimmu.2019.02923)

# *Helicobacter pylori* dampens MHC-II expression on macrophages via the up-regulation of miRNAs targeting CIITA

Gaia Codolo^#^, Marta Toffoletto^#^, Francesco Chemello, Sara Coletta, Gemma Soler Teixidor, Greta Battaggia, Giada Munari, Matteo Fassan, Stefano Cagnin^*^, Marina de Bernard^*^

^#^ contributed equally to this work.

* Co-corresponding authors

Correspondence: Stefano Cagnin, Department of Biology, University of Padua, Padua, Italy; Tel. +39.049.8276162; email: [stefano.cagnin@unipd.it](mailto:stefano.cagnin@unipd.it) and Marina de Bernard, Department of Biology, University of Padua, Padua, Italy; Tel. +39.049.8276309; email: [marina.debernard@unipd.it](mailto:marina.debernard@unipd.it)

# Supplementary Figures

**Supplementary Figure 1: Expression of CD86 and CD206 on the surface of macrophages infected or not with *H. pylori*.** Macrophages obtained from human monocytes after a 6-day differentiation with M-CSF were transferred in medium with no M-CSF and infected or not with *H. pylori* (MOI 10). After 48 h the expression of CD86 and CD206 was evaluated by flow cytometry. Data are expressed as median fluorescence intensity (MFI) ± SEM of 3 independent experiments, performed with 3 different cell preparations. Significance was determined by Student’s *t*-test. ^##^ p<0.01, calculated between untreated cells at 48 h and T_0_; **p<0.01, calculated between untreated and infected cells.





**Supplementary Figure 2: Venn diagram of differentially expressed miRNAs of macrophages infected with *H. pylori*.** Venn diagram represents the distribution of differentially expressed miRNAs.


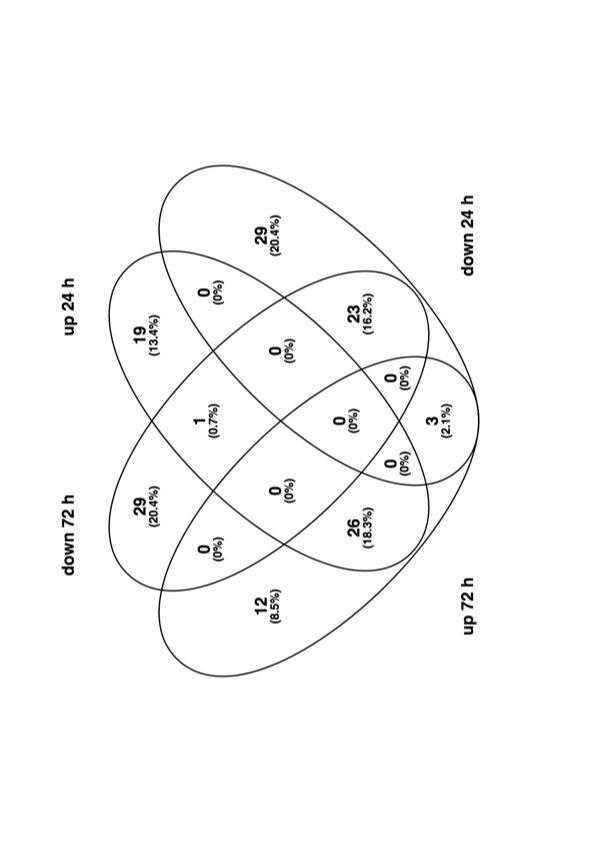


**Supplementary Figure 3: Venn diagrams of differentially expressed miRNAs in gastric cancer samples. A.** Venn diagrams represent the distribution of differentially expressed miRNAs of two gene sets (GSE23739 and GSE93415) describing miRNA expression in GC and of miRNAs altered in macrophages infected with *H. pylori*. **B.** Venn diagrams represent the distribution of differentially expressed miRNAs in one gene set (GSE26596) describing miRNA expression in GC and miRNAs altered in macrophages infected with *H. pylori*. In both cases, shared miRNAs are indicated with a color code.


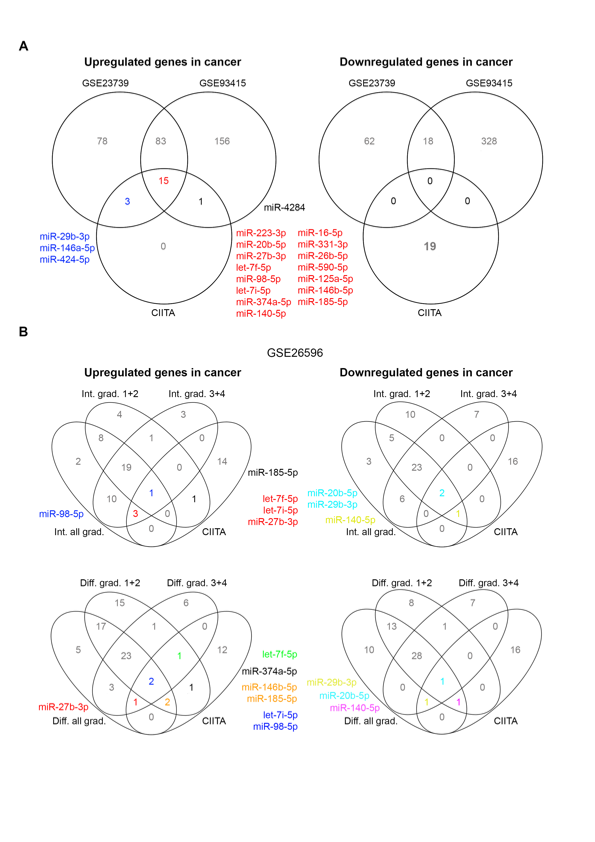


**Supplementary Figure 4: Quantification of miRNAs in transfected cells**. **A**. M121224 melanoma cells activated for 3 h with 50 ng/ml IFN-γ were transfected with pCMV-miR plasmid expressing the indicated miRNAs for 48 h. Relative expression of each miRNA was determined. Data were normalized to an endogenous reference gene (U6). Value of cells transfected with pCMV-miR plasmid expressing the miRNA scramble was taken as reference and set as 1 and the expression levels for treated cells were relative to the expression of control cells. **B.** HeLa-CIITA cells were transfected with pCMV-miR plasmid expressing the indicated miRNAs for 48 h. Data were normalized to an endogenous reference gene (U6). Values of cells transfected with pCMV-miR plasmid expressing the miRNA scramble was taken as reference and set as 1 and the expression levels for treated cells were relative to the expression of control cells.





**Supplementary Figure 5: *H. pylori* infection prevents IFN-γ-induced activation of STAT1**. Macrophages were infected with *H. pylori* (MOI 10 or 50) for 16 h and treated with IFN-γ (10 or 50 ng/ml) for 30 min. The total protein level of STAT1 and the phosphorylated form were revealed by immunoblot. Blot refers to a representative of 3 independent experiments.


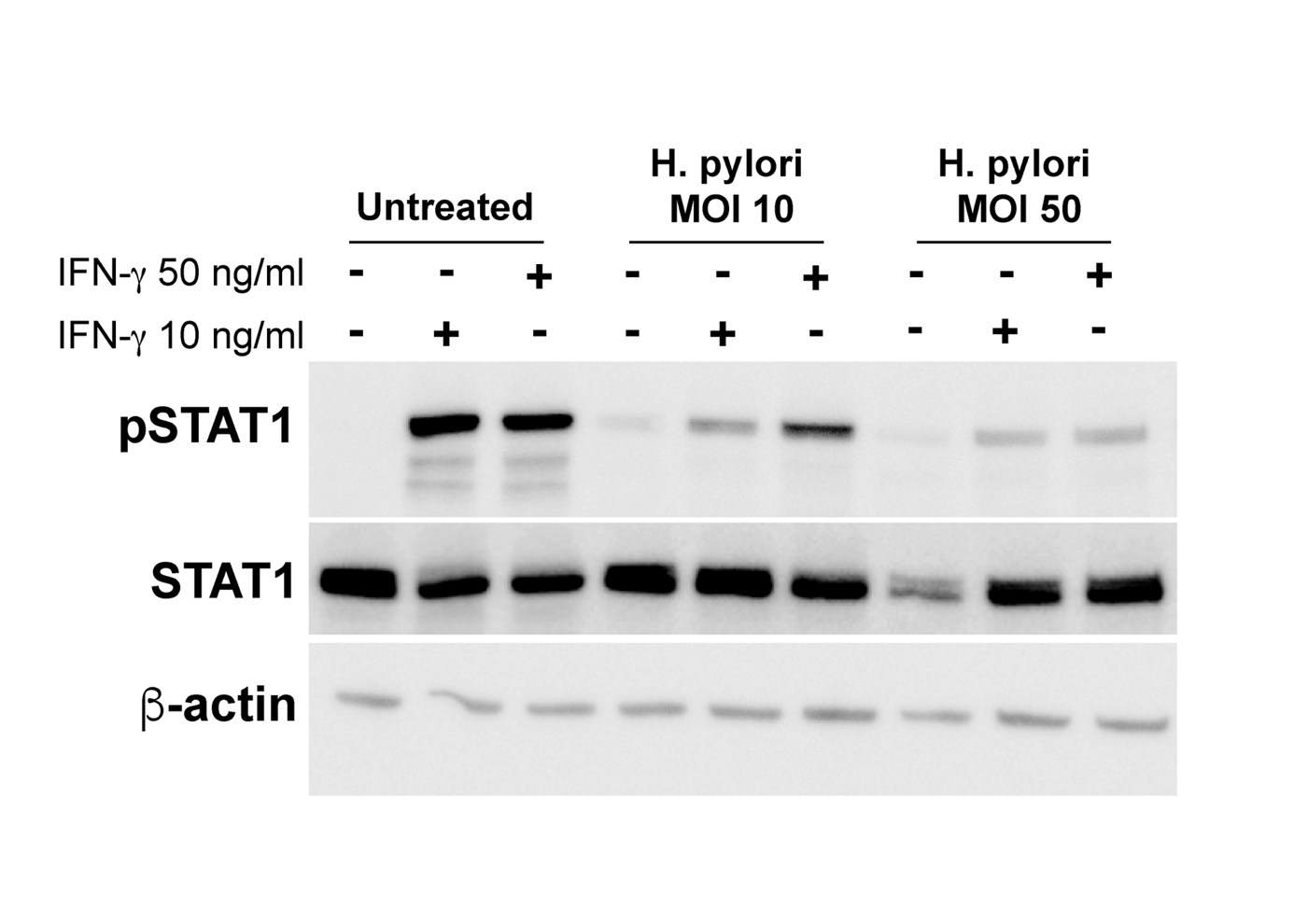


**Supplementary Figure 6: HP-NAP, VacA, the pathogenicity island CagPAI and full motility are not required for the effect of *H. pylori* on HLA-II expression in macrophages. A.** Human macrophages were infected with the indicated strains (MOI 10). For each mutant the correspondent wt strain was applied (see Materials and Methods section). Results were comparable among the different wt strains, thus data shown refer only to the P12 strain. After 24, 48 and 72 h the expression of HLA-II protein was evaluated by FACS analysis. Data are expressed as median fluorescence intensity (MFI) ± SEM of 3 independent experiments, performed with cells from 3 different donors. Significance was determined by Student’s *t*-test. ***p<0.001. **B.** HLA-II total cell content evaluated by western blot in macrophages infected as in A for 48 h. Blot refers to a representative of 3 independent experiments.


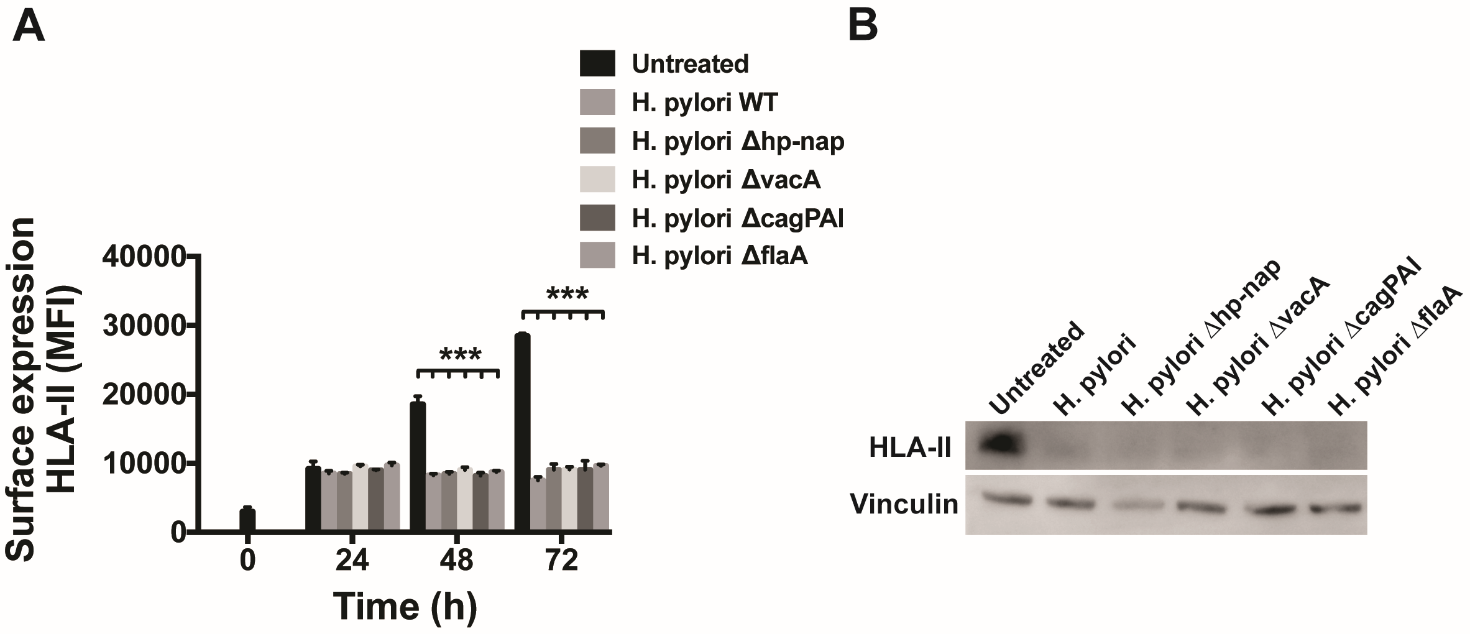

Supplement: Supplementary file 7 [file Data_Sheet_1.docx]
